# Role of Oak Ellagitannins in the Synthesis of Vitisin A and in the Degradation of Malvidin 3-*O*-Glucoside: An Approach in Wine-Like Model Systems

**DOI:** 10.1021/acs.jafc.2c00615

**Published:** 2022-04-19

**Authors:** Cristina Alcalde-Eon, María-Teresa Escribano-Bailón, Ignacio García-Estévez

**Affiliations:** Grupo de Investigación en Polifenoles, Departamento de Química Analítica, Nutrición y Bromatología, Facultad de Farmacia, University of Salamanca, E-37003 Salamanca, Spain

**Keywords:** malvidin 3-*O*-glucoside, vitisin A, oak ellagitannins, castalagin, vescalagin, degradation products, syringic acid, 2,4,6-trihydroxybenzaldehyde

## Abstract

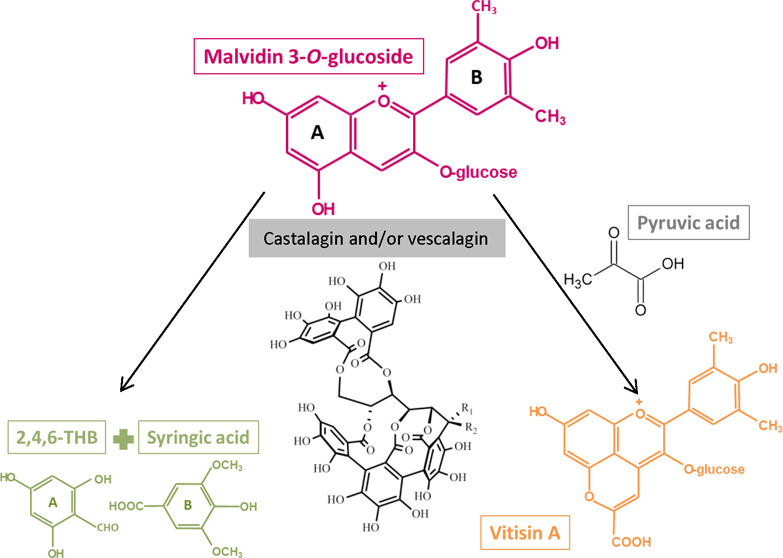

Recent studies highlight the influence that oak ellagitannins can have on wine astringency and color. Direct reactions between flavanols or anthocyanins with vescalagin have been reported to occur, but participation of these compounds in the formation of other types of derivatives has only been suggested but not demonstrated. This study aims at evaluating, in wine-like model systems, the possible different roles of the main oak ellagitannins, castalagin and vescalagin, alone or combined, in the synthesis of vitisin A and in the degradation of malvidin 3-*O*-glucoside. In the presence of pyruvic acid, the anthocyanin disappeared mainly as a result of the synthesis of vitisin A, whereas in its absence, degradation reactions prevailed. In general, ellagitannins increased the synthesis of vitisin A, decreased the total content of degradation products, and changed the degradation profile, with differences observed between castalagin and vescalagin. The results of the study revealed that the fate of malvidin 3-*O*-glucoside is conditioned by the presence of ellagitannins.

## Introduction

Anthocyanins, the main pigments of red grapes, are extracted from grapes to the must during winemaking, therefore being responsible for the color of young wines. However, the color of red wines evolves from red purple in young wines to brick red in old wines. During winemaking and aging, anthocyanins come across a large variety of compounds in solution, originating from not only grapes but also the fermentative processes and even the containers (wooden vats or barrels) where winemaking and aging are taking place. As a result, reactions between anthocyanins and these compounds may occur, causing a transformation of the coloring matter from grape native anthocyanins to anthocyanin-derived pigments. These anthocyanin-derived pigments can be formed mainly by two types of reactions. In the first type, anthocyanins react with flavanols (monomers and oligomers), either directly^1^ or mediated by acetaldehyde^2^ or other aldehydes^3^ producing flavanol–anthocyanin condensation products. In the second type of reactions, anthocyanins react with a compound having a polarizable double bond^4^ giving rise to a new pyran ring in the structure. These anthocyanin-derived pigments, named pyranoanthocyanins, importantly increase their percentages over the total pigment content as the wines become older^5,6^ and may account for more than 50% of the oligomeric coloring matter in aged wines.^6^ Among the wine compounds that can react with grape native anthocyanins to form pyranoanthocyanins, pyruvic acid, acetaldehyde, hydroxycinnamic acids, and their corresponding vinylphenols, vinylflavanols or acetoacetic acid have been widely reported.^4,5,7−11^ The maximum wavelength (λ_max_) of the ultraviolet–visible (UV–vis) spectra of these types of pyranoanthocyanins is hypsochromically shifted in relation to those of the anthocyanins from which they are formed.^4,7,10−12^ Consequently, these types of compounds express colors with more orange hues than the native anthocyanins.^4,12−14^ In addition, the new pyran ring in the structure makes the nucleophilic attack of water or bisulfite that could lead to colorless forms difficult,^12−14^ which makes the color expressed by pyranoanthocyanins more resistant against pH changes and SO_2_ bleaching. Among these compounds, vitisin A^12^ or 10-carboxypyranomalvidin 3-*O*-glucoside^11^ (formed from the reaction between malvidin 3-*O*-glucoside and pyruvic acid)^8,9^ has demonstrated to be more stable in aged wines than the native anthocyanin.^6^ In fact, as the wine ages, the percentage over the total pigment content of this anthocyanin-derived pigment increases, whereas that of malvidin 3-*O*-glucoside decreases.^6^ Schwarz and co-workers^15^ reported decreases lower than 45% in the levels of vitisin A in red wines aged for more than 10 years where malvidin 3-*O*-glucoside was no longer detectable. Comprehensive studies on red wine coloring matter^5,6,10^ have revealed that this type of pyranoanthocyanin can be formed from not only malvidin 3-*O*-glucoside but also all of the grape native anthocyanins, including acylated compounds. In addition, vitisin A can be the precursor of other oligomeric anthocyanin-derived pigments, namely, portisins, which can result from the reaction between this pyranoanthocyanin and vinylflavanols^16^ or hydroxycinnamic acids.^17^

Asenstorfer and co-workers^18^ studied the formation of vitisin A during red wine fermentation and maturation and concluded that reactive oxygen species (ROS) rather than oxygen itself were needed as oxidants to complete the synthesis of this compound. Thus, the presence of compounds favoring the formation of ROS, such as ellagitannins,^19^ might promote the synthesis of vitisin A during wine maturation. In fact, previous studies carried out in our laboratory in model systems^20^ and wines^20,21^ showed that greater levels of A-type vitisins were formed in the presence of enological tannins. In addition, in these latter model systems, the presence of the enological tannins also seemed to affect the disappearance rate of the anthocyanins.^20^ In standard wine, the anthocyanins disappeared faster in the presence of ellagitannins than in their absence, but when the precursors of vitisin A were present in the solution, the anthocyanins disappeared more slowly in the presence of ellagitannins. It is important to highlight that the enological tannins employed in these studies contained both condensed and hydrolyzable tannins (ellagitannins), making the assessment of the contribution of each type of compound to the final effect in the levels of A-type vitisins and native anthocyanins difficult. The use of simpler model systems, containing only one of these types of tannins can overcome this problem. Ellagitannins can be released from wooden containers (vats or barrels) to the wine at different stages of winemaking and aging, and among the phenolic compounds that can be extracted from wood, they seem to be the main oxygen consumers.^22^ Castalagin and vescalagin are the major ellagitannins in oak wood, whereas other *C*-ellagitannins deriving from their dimerization or the addition of a pentose in C1 of vescalagin^23^ are present in lower contents. The levels in oak wood depend upon several factors, such as the oak species or geographical origin,^23^ but their extractability from containers to wine will be the main factor determining the content in wine, which, in turn, will depend upon the manufacturing of the staves from wood and the seasoning and toasting treatments.^23−25^ Also, the number of uses and reuses of the barrels can be relevant.^26^ Although castalagin and vescalgin only differ on the conformation of C1, they show different physicochemical properties^27^ and reactivity.^28^ The objective of the present study was to evaluate the possible different roles of castalagin and vescalagin in the formation of vitisin A, in the disappearance of malvidin 3-*O*-glucoside and in the formation of its degradation products in model systems containing malvidin 3-*O*-glucoside in the presence and absence of pyruvic acid. Taking into account the results of previous studies^28^ that have shown that the presence of more than one ellagitannin in solution can influence the levels of the others, the formation of vitisin A and degradation of malvidin 3-*O*-glucoside were also evaluated in model systems containing an equimolar mixture of castalagin and vescalagin. In addition, the evolutions of the contents of these two ellagitannins were also monitored in all of the model systems.

## Materials and Methods

### Oak Wood Ellagitannins

Castalagin and vescalagin were extracted and purified from *Quercus petraea* (Matt.) Liebl. wood as described in a previous study.^29^

### Malvidin 3-*O*-Glucoside

Malvidin 3-*O*-glucoside (mv-3-glc) was obtained from *Vitis vinifera* L. cv. Tempranillo grapes following a procedure previously optimized in our laboratory for the isolation of delphinidin 3-*O*-glucoside^30^ and adapted for the isolation of mv-3-glc. To be precise, the skins were separated from grapes and extracted 3 times with 999:1 (v/v) MeOH/HCl (12 M). The extracts were gathered, evaporated in a rotary evaporator to remove MeOH, and redissolved in aqueous HCl (0.1 M, pH 1). Then, the aqueous extract was loaded onto a Sephadex LH-20 (Sigma-Aldrich, St. Louis, MO, U.S.A.) column (30 × 300 mm) and eluted with aqueous HCl (0.1 M, pH 1) (the same eluent previously used to condition the stationary phase). Malvidin 3-*O*-glucoside was the first anthocyanin to elute. The purity of mv-3-glc in the fractions was checked by high-performance liquid chromatography coupled with diode array detection and tandem mass spectrometry (HPLC–DAD–MS^*n*^), with the same equipment and methodology described below for pigment identification. Fractions with purities greater than 95% were selected, gathered, and freeze-dried to obtain a powder of mv-3-glc.

### Samples

Different model systems (Figure S1 of the Supporting Information) were prepared, in triplicate, with mv-3-glc (0.20 mM) and/or pyruvic acid (pigment/pyruvic acid ratio of 1:48) and/or ellagitannins (castalagin and/or vescalagin; pigment/ellagitannin ratio of 1:0.1; and total ellagitannin concentration of 0.02 mM) in wine-like solution [12% (v/v) absolute ethanol (VWR Chemicals, Leuven, Belgium) in ultrapure water and 0.5% (w/v) dl-tartaric acid (Sigma-Aldrich, St. Louis, MO, U.S.A.) adjusted to pH 3.2 with a 0.1 M aqueous solution of NaOH (VWR Chemicals, Leuven, Belgium)]. Model systems A, B, C, and D were prepared with pyruvic acid to study the formation of vitisin A in the absence of ellagitannins (A, control) or the presence of castalagin (B), vescalagin (C), or an equimolar mixture of both (D). Model systems E, F, and G were prepared only with pyruvic acid and with ellagitannins (E, castalagin; F, vescalagin; and G, equimolar mixture of both) at the same concentrations as those used in model systems B, C, and D, respectively, and served as control samples for the evolutions of the ellagitannin levels in these ternary model systems. Additionally, the other four model systems containing only the anthocyanin (H) or the anthocyanin with castalagin (I) or with vescalagin (J) or with a equimolar mixture of both (K) were prepared to evaluate the formation of A-type vitisins or other types of anthocyanin derivatives (B-type vitisins, for instance) in the absence of pyruvic acid.

The proportion of 1:48 between mv-3-glc and pyruvic acid was selected as a compromise proportion between that calculated from the mean concentrations reported in wine for anthocyanins and pyruvic acid (proportion of 1:5)^31^ and the molar ratios employed in previous studies^9,32^ for the synthesis of A-type vitisins from skin extracts in excess of pyruvic acid (molar ratios of pyruvic acid/total anthocyanins next to 200). The proportion of 1:0.1 anthocyanin/ellagitannin was selected taking into account those employed in previous studies carried out in our laboratory.^20^ Model systems were kept in the dark and the presence of air,^32^ which is a requirement for the oxidation step occurring in a late stage of the synthesis.^18^ A temperature of 23 °C was selected on the basis of the results reported by Romero and Bakker.^9^

Model systems were monitored by HPLC–DAD–MS^*n*^ for 122 days. For this purpose, samples aiming at evaluating the disappearance of anthocyanin and the appearance of vitisin A over time were taken at days 1 (just after the preparation of the model systems), 3, 5, 9, 12, 17, 21, 28, 35, 46, 63, 77, 97, and 122 (end of the study). The study of the evolution of the ellagitannin samples was performed at days 1, 5, 12, 21, 35, 46, 77, 97, and 122.

### Analysis of Pigments by HPLC–DAD–MS^*n*^

Prior to the injection into the HPLC–DAD–MS^*n*^ system, samples were diluted 1/2 with acidified water and filtered through a 0.45 μm Millex syringe-driven filter unit (Millipore Corporation, Bedford, MA, U.S.A.). A Hewlett-Packard 1100 series liquid chromatograph (Agilent Technologies, Waldbronn, Germany) was connected via the cell outlet to an API 3200 Qtrap mass spectrometer (Applied Biosystems, Darmstadt, Germany) equipped with an electrospray ionization (ESI) source and a triple quadrupole ion trap mass analyzer and controlled by Analyst 5.1 software. The chromatographic column was an AQUA C18 reversed-phase, 5 μm, 150 × 4.6 mm column (Phenomenex, Torrance, CA, U.S.A.) thermostated at 35 °C. Eluents were an aqueous 0.1% trifluoracetic acid solution (solvent A) and HPLC-grade acetonitrile (solvent B). HPLC–DAD conditions were previously optimized in our laboratory^5^ and allowed the complete separation between the peaks corresponding to malvidin 3-*O*-glucoside, vitisin A, vitisin B, and 10-methylpyranomalvidin 3-*O*-glucoside. The value of 520 nm was selected as the preferred wavelength for the chromatograms, and spectra were recorded from 220 to 600 nm. Pigments were quantified by means of a calibration curve of mv-3-glc (Extrasynthese, Genay, France). MS analyses were performed in positive ion mode (ESI^+^), and the settings were previously optimized by direct infusion of a malvidin 3-*O*-glucoside solution.^21^ Zero-grade air served as nebulizer gas (GS1) and turbo gas (GS2) for solvent drying. Nitrogen served as curtain (CUR) and collision gas (CAD). The mass method consisted of three mass experiments (full mass (EMS mode), MS^2^, and MS^3^ analyses). Spectra were recorded between *m*/*z* 150 and 1100.

### Analysis of Ellagitannins by HPLC–MS^*n*^–Multiple Reaction Monitoring (MRM)

Samples were also diluted 1/2 with acidified water prior to the analysis of ellagitannins. Then, following the methodology developed and validated in our laboratory,^33^ (−)-gallocatechin (Sigma-Aldrich, St. Louis, MO, U.S.A.) was added as an internal standard (final concentration of 0.015 mg/mL) and the samples were filtered (0.45 μm Millex syringe-driven filter unit) before the injection in the HPLC–MS system. A Hewlett-Packard 1200 series LC equipped with an AQUA C18 reversed-phase, 5 μm, 150 × 4.6 mm column (Phenomenex, Torrance, CA, U.S.A.) thermostated at 35 °C was employed, using an aqueous solution (2.5%) of acetic acid (AnalaR, Normapur, VWR International, Fontenay-sous-Bois, France) (solvent A), 100% HPLC-grade isopropanol (HiPerSolv Chromanorm, BDH Prolabo, VWR International, Fontenay-sous-Bois, France) (solvent B), and 100% HPLC-grade methanol (Macron Fine Chemicals, Avantor, Gliwice, Poland) (solvent C) as eluents. HPLC conditions were previously developed in our laboratory for the analysis of oak wood ellagitannins.^29^ The API 3200 QTrap mass spectrometer was connected to the LC via the cell outlet, and MS^*n*^–MRM analyses were performed in negative ion mode following the conditions previously optimized for quantification of these compounds.^33^

### Statistical Analysis

Tukey’s honestly significant difference test (*p* < 0.05) was performed with the IBM-SPSS Statistics 23 for Windows software package to evaluate the significance of the differences observed among samples. Results of the statistical analysis are included in the tables shown in the Supporting Information.

## Results

### Evolution of the Pigments

**Figure 1:**
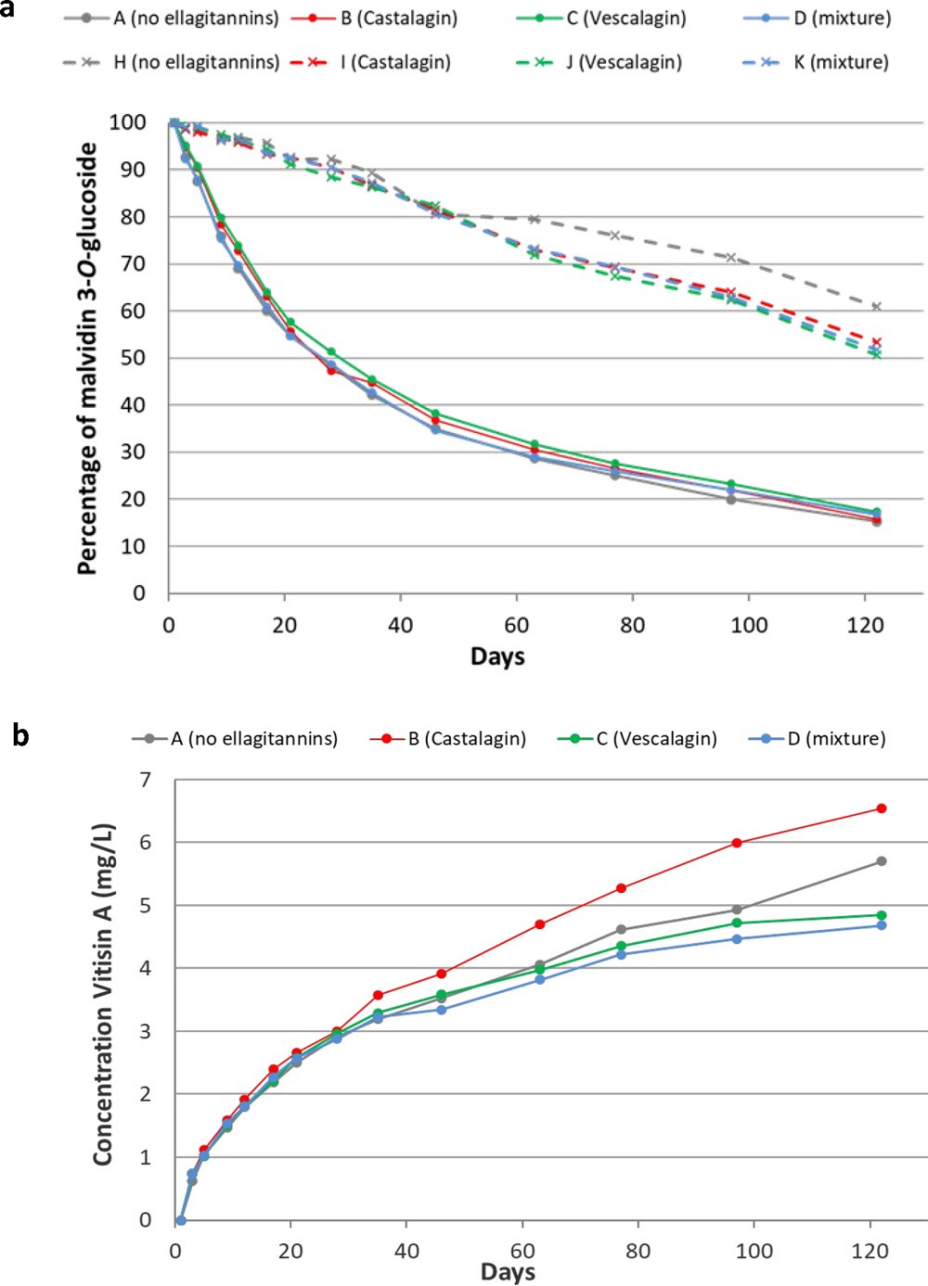
(a) Evolution of the levels of malvidin 3-*O*-glucoside in model systems A, B, C, and D (with pyruvic acid) and H, I, J, and K (without pyruvic acid) expressed as percentages over the initial concentration in each model system. (b) Evolution of the levels of vitisin A (expressed in mg/L of malvidin 3-*O*-glucoside) in the model systems containing pyruvic acid (model systems A, B, C, and D). Ellagitannin present in each model system is indicated in parentheses in the legend. See Tables S1 and S2 of the Supporting Information for the statistical significance of the differences observed.

The evolution of the content of mv-3-glc was studied as a percentage of the initial levels at day 1 (Figure 1a; see Table S1 of the Supporting Information for the statistical significance of the observed differences). An important decrease of the initial content could be observed during the length of the study, meaning that transformation and/or degradation of mv-3-glc was occurring. However, a different evolution was observed between the model systems containing pyruvic acid (A, B, C, and D) and those where it was absent (H, I, J, and K). In the former model systems, the levels followed an exponential evolution, with a faster decrease during the first 28 days and with a slower decrease from then onward. In contrast, the evolution of mv-3-glc in the latter model systems was almost linear (Figure 1a) and slower than that of the former during the first days of the experiment. Consequently, at day 28, only 50% of the initial content of anthocyanin remained in solution in models A–D, whereas in models H–K, it still represented about 90%. In the last part of the experiment, model system H showed the lowest decrease in the levels (Figure 1a and Table S1 of the Supporting Information), which can be explained by the absence of pyruvic acid and ellagitannins and, consequently, by the limited occurrence of reactions of mv-3-glc related with these compounds. Despite this difference in the levels, the behavior of mv-3-glc in this model system was similar to those observed in all of the models that contained ellagitannins in the absence of pyruvic acid (model systems I, J, and K). Therefore, it seems that, in the absence of pyruvic acid, ellagitannins are, above all, increasing the rates of the reactions occurring in the model systems only containing anthocyanin (either degradation or transformation reactions). In the presence of pyruvic acid (model systems A–D), the faster decreasing rate and the nonlinear behavior were pointing to the occurrence of new reactions involving anthocyanin and pyruvic acid. In a previous study carried out in more complex model systems,^20^ it was reported that the disappearance of anthocyanins was much faster in the model systems prepared in a standard medium resulting from the fermentative metabolism of glucose than in those prepared in wine-like solution, and that in the former model systems, anthocyanin-derived pigments, such as A- and B-type vitisins, were formed. Furthermore, as occurred in the present study in model system H, the lowest disappearance of anthocyanins took place in the model system prepared in wine-like solution and the absence of enological tannins. In contrast, among the model systems prepared with pyruvic acid (model systems A–D), the lowest disappearance of mv-3-glc occurred in those containing a single ellagitannin [castalagin (B) or vescalagin (C)] and the greatest disappearance could be observed in that where ellagitannins were absent (model system A) and also in that containing an equimolar mixture of them (model system D) (Table S1 of the Supporting Information). The presence of a single ellagitannin in the model systems containing pyruvic acid, therefore, seems to reduce the disappearance rate of mv-3-glc and might be pointing to a protective role of these ellagitannins against degradation.

Taking into account these first results on mv-3-glc disappearance that point to a possible influence of the tested ellagitannins in the degradation of mv-3-glc and/or in the formation of anthocyanin-derived pigments, the samples were monitored to evaluate the role of the main oak ellagitannins in the formation of the main degradation products (see the Evolution of the Degradation Products of Malvidin 3-*O*-Glucoside section below) and in the synthesis of mv-3-glc-derived pigments.

With regard to the derivative pigments, the synthesis of vitisin A was possible in model systems A, B, C, and D because of the presence of pyruvic acid. Additionally, the fact that all of the model solutions were prepared with ethanol and that they were kept in the presence of air might make the formation of acetaldehyde and, therefore, the synthesis of vitisin B possible in all of the models containing mv-3-glc (model systems A–D and H–K). The analyses of the new peaks appearing in the chromatograms during the experiment confirmed that the synthesis of vitisin A but not that of vitisin B was taking place in model solutions A–D. In model systems H–K, only vitisin B could be detected but at trace levels [extracted ion chromatogram (XIC) at *m*/*z* 517], which made the evaluation of the influence of ellagitannins in its formation impossible. This restricted synthesis of vitisin B in the absence of acetaldehyde but in the presence of its precursors (ethanol in the presence of air and oxidants, such as ellagitannins) was also observed in more complex model systems^20^ and supports the evolution of the levels of B-type vitisins reported in wines,^5,6^ with greater contents at earlier stages, when acetaldehyde coming from fermentation is available, and lower contents at late stages, when acetaldehyde is mainly formed from the oxidation of ethanol. During the experiment, a new peak corresponding to 10-methylpyranomalvidin 3-*O*-glucoside also appeared in model systems A–D, and its evolution was also monitored. Taking into account that anthocyanins and ellagitannins can react to form anthocyanoellagitannins^30,34,35^ and that this reaction only occurs with ellagitannins possessing the substituent at C1 in β orientation, model systems containing vescalagin (C, D, J, and K) were also monitored in search of the derivative compound between mv-3-glc and vescalagin. Several XIC analyses were carried out in the results from the mass analyses in positive and negative mode at *m*/*z* of this derivative pigment (M^+^ or [M – H]^−^), at *m*/*z* of its aglycone ([M^+^ – 162] or [M – H – 162]^−^), or at *m*/*z* of its doubly charged ion, but no signals could be observed in any of the model systems.

In the case of vitisin A (Figure 1b; see Table S2 of the Supporting Information for the statistical significance of the observed differences), it was detectable in model systems A–D from day 3 of the experiment. Its concentration increased during the whole experiment, with the fastest rate being observed during the first days. The model system containing castalagin (model system B) showed the greatest content in most of the sampling points. This result is in agreement with those reported in more complex model systems,^20^ where samples added with enological tannins that contained ellagitannins exhibited greater contents than those prepared without them. Because a late step of oxidation is required to complete the synthesis of vitisin A,^18^ castalagin might be acting as an oxidant, boosting the synthesis of vitisin A. In contrast, the levels determined in those containing vescalagin either alone (model system C) or combined with castalagin (model system D) were not statistically different from those observed in the absence of ellagitannins (model system A), highlighting differences in the behavior between castalagin and vescalagin. This reduced rate of vitisin A synthesis in vescalagin-containing model systems (C and D) was observable, above all, from day 35 and might be associated, on the one hand, to the lower levels of vescalagin available in these model systems (see below) in relation to those of castalagin, despite having the same initial total concentration. This faster disappearance of vescalagin than castalagin has also been reported in aqueous solution in both the absence and presence of air^28^ and is in agreement with the greater reactivity reported for it.^27,35^ In addition, in model system D, the presence of more than one ellagitannin in solution caused, as previously reported,^28^ an important reduction of the levels of each ellagitannin in relation to those observed when only a single ellagitannin was present (see the Evolution of Ellagitannins section below). Thus, in these model systems (C and D), the availability of the oxidant necessary to complete the synthesis would be reduced and, consequently, the levels of vitisin A. On the other hand, the greater reactivity of vescalagin might be increasing at the same time in turn, the rate of reactions causing the transformation and/or degradation of vitisin A. Thus, the concurrence of a lower synthesis and a greater transformation of vitisin A might explain the steady state observed in the levels of vitisin A in model systems C and D at the end of the study. These results highlight the role of ellagitannins as oxidants during the synthesis of vitisin A and the relevance of the types and proportions of these compounds in their oxidative role.

Similar to vitisin A, 10-methylpyranomalvidin 3-*O*-glucoside (Figure S2 of the Supporting Information) was also detected in the model systems containing pyruvic acid (A, B, C, and D) but at much lower concentrations (about 50 times lower). Its content also increased during the experiment but showed a more linear evolution than vitisin A. As in the case of vitisin A, the greatest concentration was observed in the model system exclusively containing castalagin (model system B), whereas those containing vescalagin (C and D) behaved similar to model system A, where no ellagitannins were added. This anthocyanin-derived pigment has been identified in different types of red wines,^5,36^ and it can be originated from the reaction of mv-3-glc with either acetone^5^ or acetoacetic acid.^36^ In the present study, the origin of this compound still remains unclear, because none of these two precursors were initially present in the model systems where it was detected (they only contained mv-3-glc and pyruvic and tartaric acids in the presence of ethanol). Given the similarity between the behaviors of this compound and vitisin A, it might be proposed that an oxidation step is also required for its synthesis, which would also depend upon the levels of the oxidants present in solution. In addition, ellagitannins and, above all, castalagin, as a result of its greater levels, might be favoring the transformation of the acids present in the model systems into the precursors, acetone or acetoacetic acid, which would react with mv-3-glc for the origination of the pyranoanthocyanin compound.

### Evolution of the Degradation Products of Malvidin 3-*O*-Glucoside

**Figure 2:**
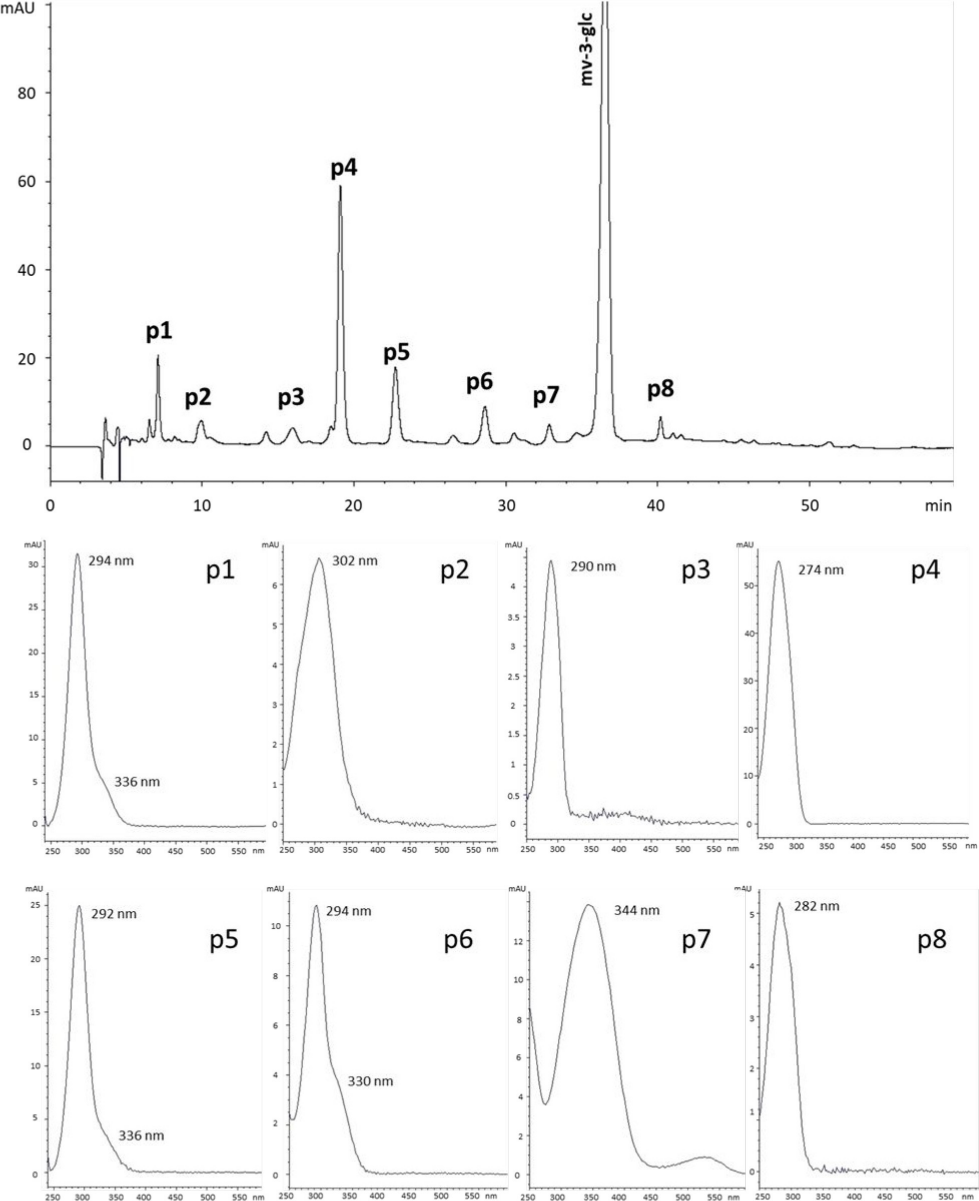
Chromatogram corresponding to model system H at day 122, recorded at 280 nm with the chromatographic conditions for the analysis of pigments (upper part of the figure). UV–vis spectra and absorption maxima of the eight degradation compounds of mv-3-glc detected in the sample (compounds **p1**–**p8**) (lower part of the figure). See Table S3 of the Supporting Information for UV and MS spectral features and identities proposed for the different compounds.

As mentioned above, the disappearance of mv-3-glc occurred in all of the model systems containing anthocyanin, even in those where transformation reactions were limited (model systems H, I, J, and K). This reduction in the levels of mv-3-glc without formation of anthocyanin-derived pigments was pointing to the occurrence of degradation reactions. Model system H served as a control sample for the study of the degradation products of mv-3-glc, because it only contained anthocyanin dissolved in wine-like solution. Eight peaks that might correspond to degradation products of mv-3-glc were detected in this model system at the end of the study (Figure 2 and Table S3 of the Supporting Information).

Among these eight peaks, peak **p4** was the most abundant (43.3% of the total degradation products), followed by peak **p5** (17.5%). On the basis of the results of previous studies about degradation products of mv-3-glc^37−39^ and taking into account the UV and mass spectral features of peaks **p4** and **p5** (Figure 2 and Table S3 of the Supporting Information), they were identified as syringic acid and 2,4,6-trihydroxybenzaldehyde (THB), respectively. These two compounds have been reported to be the main degradation products of mv-3-glc and malvidin 3,5-*O*-diglucoside (mv-3,5-diglc) under oxidative conditions or after thermal, enzymatic, or microwave treatments^38−40^ originating from the B ring (syringic acid) and A ring (THB). Different mechanisms of formation have been proposed,^40−42^ but all of them involve the hydrolysis of glycosidic bonds, the opening of the pyrylium ring, and the cleavage of chalcone. Differences concern, above all, the order of occurrence of deglycosylation and opening of the C ring^39,40^ and might be related to the pH, as reported for cyanidin 3-*O*-sophoroside.^42^ At pH next to 1, deglycosylation is the first step, whereas at pH next to 3.5, it is the opening of the C ring, making the detection of chalcone 3-*O*-glucoside in the sample possible. In our samples, prepared at pH 3.2, chalcone 3-*O*-glucoside of malvidin (peak **p7**; Figure 2) could be detected and identified from its UV–vis features (344 nm; Figure 2) and mass spectra features (molecular ion at *m*/*z* 511 and fragment ion at *m*/*z* 349; Table S3 of the Supporting Information). Thus, it seems that, in the present study, compounds **p4** and **p5** are mainly formed by the mechanism proposed by Zhao and co-workers,^39^ with the opening of the pyrylium ring being the first step. Peak **p6** was the third most abundant peak (9.8%). Its UV (λ_max_ at 294 nm; shoulder around 330 nm; Figure 2) and mass spectral (molecular ion at *m*/*z* 335; fragment ion at *m*/*z* 181; Table S3 of the Supporting Information) data were in accordance with the features of a compound previously reported by Lopes and co-workers^38^ in the thermal degradation of mv-3-glc, whose structure could not be completely elucidated. From the mass results and fragmentation pattern, these authors concluded that this compound still contained in its structure the two aromatic rings of malvidin, but without the glucose moiety, and that the resulting fragment ion was formed by the loss of THB. Although the mechanism of formation was not reported, it was proposed to be an intermediary product in the formation of syringic acid and THB from mv-3-glc.^38^ More recently, Vallverdú-Queralt and co-workers^43^ also detected this compound in model solutions containing mv-3-glc and proposed an structure for it. According to that study,^43^ compound **p6** might be formed as an intermediary product in the transformation of malvone aglycone to THB. Peak **p1** showed an UV spectrum very similar to that of THB (peak p5; Figure 2), but it eluted earlier than it, pointing to the presence of substituents conferring more polarity. Taking into account that its molecular ion showed 16 additional amu (*m*/*z* 171) in relation to peak **p5**, compound **p1** was proposed to be 2,4,6-trihydroxybenzoic acid. Its presence might be related to the oxidation of THB because the model systems were prepared and maintained in the presence of air. In fact, Piffaut and co-workers^40^ have reported the formation of phloroglucinol from THB in drastic thermal conditions (100 °C for 15 h) through the oxidation of the latter to the corresponding acid and then by decarboxylation of the acid. Because the conditions were not so drastic in the present study, phloroglucinol was not detected in any of the sampling points. Instead, 2,4,6-trihydroxybenzoic acid was detected from the first sampling point until the end of the experiment, although always in levels lower than THB. Peak **p8** was proposed to be syringaldehyde based on its UV spectrum (similar to that of syringic acid but with a bathochromic shift of 8 nm in its λ_max_; Figure 2) and the signal at *m*/*z* 183 observed in its mass spectrum. However, its mechanism of formation still remains unclear. Reports on its presence are scarce,^39^ and formation in oxidative media such as those of the present study is unlikely to occur from syringic acid. Peaks **p2** and **p3** were the least abundant peaks in most sampling points of the experiment in model system H (<5%). As a result of their low contents, no mass spectrometric data were available for peak **p2** and only the *m*/*z* ratio of the protonated molecular ion (*m*/*z* 169) and not those of the fragment ions could be obtained for peak **p3**. The UV spectra of these compounds were available (λ_max_ of peak **p2** at 302 nm and λ_max_ of peak **p3** at 290 nm; Figure 2), and although they did not allow for their complete identification, they supplied useful information in the case of peak **p3**. To be precise, the UV spectrum ruled out the possibility for this compound to be vanillic acid (*m*/*z* 169), the main degradation product of peonidin 3-*O*-glucoside, which was present as an impurity of mv-3-glc (lower than 2% of the total area at 520 nm) in the samples.

**Figure 3:**
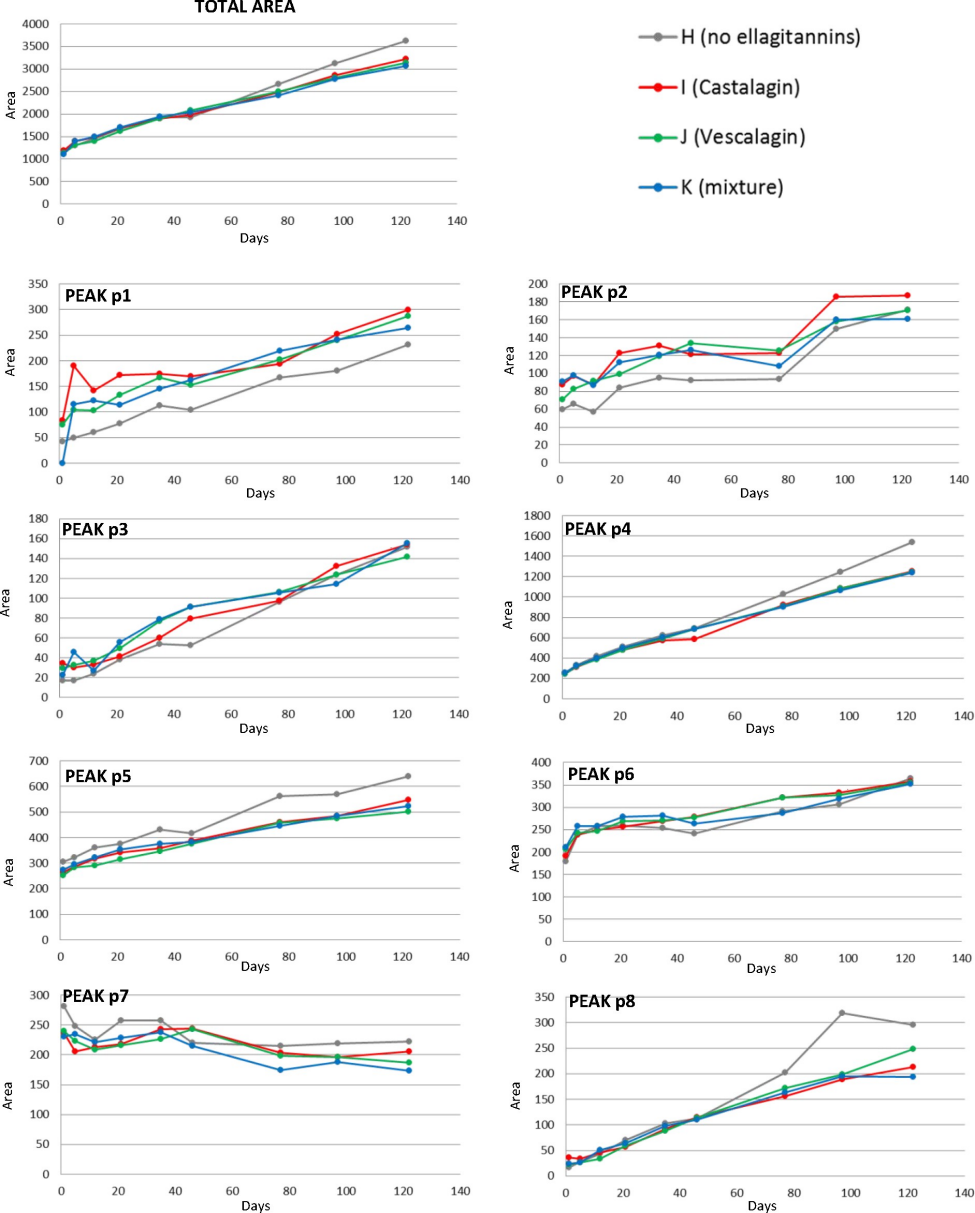
Evolution over time of the mean (*n* = 3) total and individual levels of the main degradation products of malvidin 3-*O*-glucoside in model systems H, I, J, and K. See Table S4 of the Supporting Information for significance of the differences among model systems at each sampling point.

All of these degradation products were monitored from days 1 to 122 in not only model system H but also those containing ellagitannins (model systems I, J, and K), to study the influence that the presence of one or more ellagitannins might have on their evolution. The total content of these degradation products increased from day 1 until the end of the experiment in all of the model systems (Figure 3 and Table S4 of the Supporting Information). However, the evolution was not the same for all of the compounds detected (Figure 3 and Table S4 of the Supporting Information), which might be informative about different mechanisms of formation. Compound **p7** (chalcone 3-*O*-glucoside) was the only compound whose levels tended to decrease during the first sampling points and then stabilize. This is in accordance with the mechanism proposed above, where chalcone 3-*O*-glucoside is an intermediate in the formation of compounds **p4** and **p5**. Thus, as long as mv-3-glc is available, chalcone 3-*O*-glucoside can be formed, which, in turn, can be deglycosylated and cleaved to form compounds **p4** and **p5** in a final step, therefore explaining the stabilization of its levels. The levels of the rest of the compounds tended to increase but at different rates (Figure 3), which can be informative if the compounds are end products of the main degradation pathway and tend to accumulate or if they are intermediates or end products of secondary pathways, whose levels depend upon the equilibrium between their formation and their transformation into other products. With regard to the influence of ellagitannins in the formation of these degradation compounds, differences were observed among compounds. Compounds **p1** and **p2** showed greater levels in the model systems containing ellagitannins (I, J, and K) than in their absence (model system H) (Figure 3). This observation, as occurred in the case of the synthesis of vitisin A, is pointing to the oxidative nature of the reactions leading to the formation of these compounds, where ellagitannins, through their reaction with oxygen and formation of *ortho*-quinones,^44^ can be favoring them. For the formation of these two compounds (**p1**, and **p2**) and compound **p6**, the type or number of different ellagitannins in the model system was not as relevant as for the synthesis of vitisin A. In the particular case of compound **p1**, the greater levels observed in the presence of ellagitannins confirm the hypothesis that compound **p1** may originate from the oxidation of compound **p5**. Compound **p6** also showed greater levels in the presence of ellagitannins, but differences were significant only in the middle part of the experiment, which is in accordance with the intermediary nature proposed for it. Contrary to these three compounds (**p1**, **p2**, and **p6**), compounds **p4** and **p5** (the most abundant mv-3-glc degradation products) were produced in lower amounts in the model systems that contained ellagitannins, pointing to a lower dependence of the last steps of mv-3-glc degradation on oxidative reactions. Because these two compounds represent around 60% of the total degradation product content, it is understandable that, at the end of the experiment, the total levels were also lower in the presence of ellagitannins (Figure 3). In the case of compounds **p3**, **p7**, and **p8**, no clear relationship of their levels with the presence or absence of ellagitannins could be observed.

It is important to remark that model system H was the model system that, at the end of the experiment, showed the lowest decreases in the levels of mv-3-glc (Figure 1a). However, the content of degradation products was greater in that model system than in those containing ellagitannins (Figure 3 and Table S4 of the Supporting Information). This means that, in the presence of ellagitannins, some of the mv-3-glc degradation reactions are reduced and that a greater proportion of anthocyanin is devoted to the formation of derivative pigments (mainly vitisin B, as previously indicated).

As a result of the different formation rates observed for the different compounds, differences in their percentages over the total content were detected among sampling points and model systems (Figure S3 of the Supporting Information). In general, the percentage of compounds **p1** (2,4,6-trihydroxibenzoic acid), **p2**, **p4** (syringic acid), and **p8** increased over time; those of compounds **p5** (THB), **p6**, and **p7** (chalcone 3-*O*-glucoside) decreased; and that of compound **p3** increased during the first sampling points and then decreased until the end of the experiment. As observed for the content, the percentages of these eight degradation products were influenced by the presence of ellagitannins, with those of compounds **p1**, **p2**, and **p6** increasing and those of compounds **p4** and **p5** decreasing in the presence of ellagitannins, thus modifying the degradation product profile.

To evaluate if the presence of pyruvic acid also affects the degradation reactions of mv-3-glc, the eight degradation products detected in models H–K were also monitored in model systems A, B, C, and D. At day 122 (Table S4 of the Supporting Information), the total content was slightly lower in model systems A–D than in those where pyruvic acid was absent (H–K), meaning that degradation reactions occur to a lower extent in the presence of transformation reactions. By comparison of the levels of the different compounds in model systems where ellagitannins were absent (A and H), differences mainly as a result of the presence of pyruvic acid could be assessed. Compound **p3** was not detected in model system A; compounds **p1**, **p4**, and **p7** (2,4,6-trihydroxybenzoic acid, syringic acid, and chalcone 3-*O*-glucoside) showed lower contents than in model system H; and compounds **p2** and **p8** showed greater levels. Despite these changes in the content, the percentage (Figure S3 of the Supporting Information) of the main compounds (**p4**, **p5**, and **p6**) hardly changed between model systems A and H, with differences occurring mostly in minor compounds (greater percentages of compounds **p2** and **p8** and lower percentages of compounds **p1**, **p3**, and **p7**). With regard to the effect of the presence of ellagitannins in the model systems containing pyruvic acid (Table S4 of the Supporting Information), differences were observed between castalagin and vescalagin. At day 122, model system B (containing castalagin) showed an increase in the levels of most of the degradation compounds in relation to those observed in model system A. On the contrary, in that containing vescalagin (model system C), the formation of the main degradation compounds (**p4** and **p5**) was lower than in model system A, and those of the rest of the compounds showed increases in relation to model system A but lower than with castalagin. As commented above for vitisin A, this difference might be due to the greater reactivity of vescalagin, which can cause its earlier depletion in relation to castalagin, making it less available during the last stages of the experiment. In addition, the different reactivity of castalagin and vescalagin^27,35^ might be causing a different affinity of each ellagitannin toward the different reactions taking place, thus explaining the different effects observed for the main degradation compounds.

### Evolution of Ellagitannins

**Figure 4:**
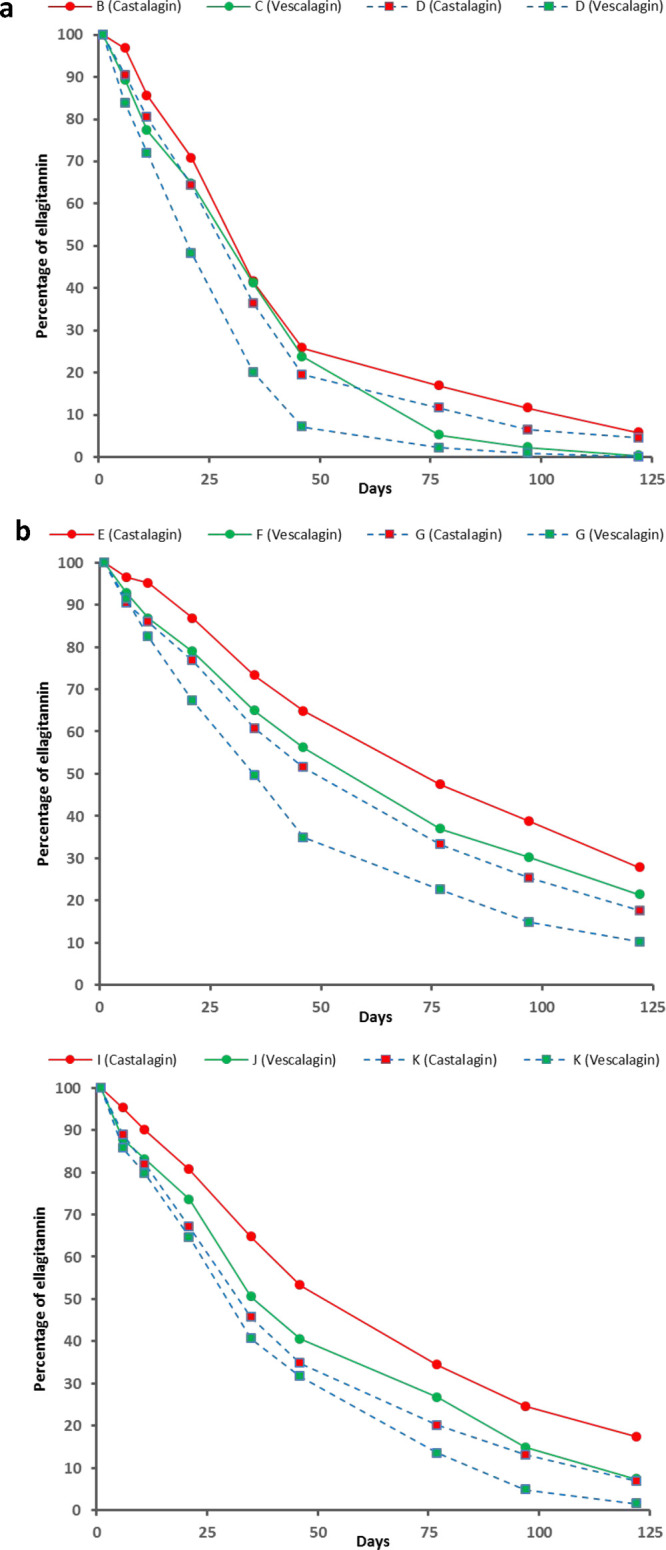
Evolution of the levels (expressed as percentages of the initial content) of the different ellagitannins (indicated in parentheses in the legend) in the (a) model systems containing ellagitannins, anthocyanins, and pyruvic acid (model systems B, C, and D), (b) model systems containing ellagitannins and pyruvic acid (model systems E, F, and G), and (c) model systems containing ellagitannins and anthocyanins (model systems H, I, and J). See Table S5 of the Supporting Information for the statistical significance of the differences observed.

Previous studies in simple model systems have revealed that ellagitannins in solution progressively disappear in not only ethanol solutions^45^ but also ultrapure water solutions^28^ as a result of hydrolysis, oxidation, or transformation reactions of the original ellagitannins. The occurrence of this disappearance, even in the absence of oxygen, points to the existence of oxygen-independent reactions, which can be boosted, in turn, by oxygen-dependent reactions.^28^ In addition, in the case of ethanol solutions, the formation of ellagitannin derivatives with ethoxy moieties added to the structure^45^ or the formation of the β-1-O-ethylated derivative from vescalagin^34,46^ can also be responsible for the reduction in the levels over time. Figure 4 shows the evolution over time of castalagin and vescalagin in the different model systems (see Table S5 of the Supporting Information for significance of the differences). In all of the cases, the levels of ellagitannins decreased over time but the rates were different, with the greatest decreases being observed in the model systems additionally containing mv-3-glc and pyruvic acid (B, C, and D), the smallest decreases in those only containing pyruvic acid (E, F, and G), and the intermediate decreases in those only containing anthocyanin (I, J, and K). These different rates are indicative of the occurrence of different types of reactions in each of them. The greater rates occurring in the model systems containing ellagitannins and mv-3-glc (with or without pyruvic acid; B, C, D, I, J, and K) in relation to those only containing ellagitannins and pyruvic acid were indicative of a participation of ellagitannins in reactions with anthocyanins. In model systems B, C, and D, the decreases in the levels of anthocyanin and ellagitannin(s) were related to the transformation of mv-3-glc into vitisin A. In contrast, in the absence of pyruvic acid (model systems I, J, and K), the disappearance of anthocyanin and ellagitannin(s) should be related above all to degradation reactions. Taking into account that the extent of the degradation reactions of mv-3-glc was quite similar in all of the model systems containing mv-3-glc, the greater disappearance of ellagitannins in the model systems containing pyruvic acid and mv-3-glc can be indicating that the reactions leading to the synthesis of vitisin A consume more ellagitannins than those leading to the degradation products. The consumption of ellagitannins was the lowest in the model systems exclusively containing ellagitannins in the presence of pyruvic acid (model systems E, F, and G), in which their disappearances could be attributed mainly to their degradation, because no important peaks corresponding to possible ellagitannin-derived compounds could be observed (commented below). Interestingly, the disappearance rates observed for castalagin and vescalagin in these model systems (E, F, and G) were slower than those observed for these ellagitannins in simpler model systems prepared in ultrapure water under an oxidative or an inert atmosphere.^28^ In those simpler model systems, where there were exclusively ellagitannins, their disappearance was mostly due to their degradation, which was, in turn, boosted by the compounds formed. In the present study, the slower disappearance might be first related to the smaller concentration of ellagitannins employed in the preparation of the model systems (circa 20 mg/L versus 40 mg/L^28^) and also to the presence of other compounds (ethanol and pyruvic and tartaric acids), which have probably “buffered” the reactions in cascade, leading to the autodegradation of ellagitannins (a lower amount of ellagitannins is available to take part in reactions, and in addition, ellagitannins can react with compounds other than themselves, reducing their participation in autodegradation reactions).

For the same types of model systems, vescalagin always showed greater losses and faster disappearance rates than castalagin, which again supported the greater reactivity reported for the former in relation to the latter.^27,28,35,45^ Thus, at the end of the experiment, castalagin accounted for almost 6, 28, and 18% of the initial content in model systems B, E, and I, whereas vescalagin disappeared almost completely in model system C (<0.4% of the initial content) and accounted for 21 and 7% in model systems F and J.

The different reactivity of vescalagin and castalagin could be observed in not only the rates of disappearance but also the evolution of their levels in the different types of model systems. In the model systems with pyruvic acid and mv-3-glc, the behaviors and levels of castalagin (model system B) and vescalagin (model system C) were quite similar until day 46, pointing to a similar participation of both ellagitannins in the last step of the synthesis of vitisin A. However, from then onward, the levels of vescalagin importantly decreased (Figure 4a). At this sampling point, mv-3-glc, the main precursor of vitisin A, was still available in both model systems, showing similar percentages (almost 40% of the initial concentration remained in solution; Figure 1a) and identical evolution until the end of the experiment. This means that the same amount of mv-3-glc was consumed for the synthesis of vitisin A in both model systems. Thus, the greater reduction in the levels observed in vescalagin from day 46 cannot be associated with a greater synthesis of vitisin A. On the contrary, as previously indicated, the amounts of vitisin A increased more slowly in model system C than in model system B from this day onward (Figure 1b). It seems, therefore, that, from day 46, vescalagin might be taking part in reactions other than the synthesis of vitisin A, including reactions that might affect the already synthesized vitisin A. Nevertheless, these differences between the levels of castalagin and vescalagin (Figure 4a and Table S5 of the Supporting Information) were lower than those observed in the model systems containing ellagitannins and pyruvic acid (model systems E and F; Figure 4b and Table S5 of the Supporting Information) or ellagitannins and mv-3-glc (model systems I and J; Figure 4c and Table S5 of the Supporting Information), which, in turn, were observable during the whole experiment (panels b and c of Figure 4). The greater decrease of vescalagin was indicative of a greater participation of this ellagitannin in other types of reactions. However, in the case of model systems I and J, where the degradation of mv-3-glc was the main reaction occurring, no important differences were observed according to the type of ellagitannin (see above), making the confirmation of a greater participation of vescalagin in this reaction difficult. In the case of model systems E and F, which only contained ellagitannins in the presence of pyruvic acid, samples were carefully checked in search of ellagitannin-derived compounds that might explain the greater losses of vescalagin in relation to castalagin. Products that might be formed from the direct reaction between pyruvic acid and ellagitannins were first searched, but no signals appeared at the expected *m*/*z* (1003, negative ion mode) in any of the model systems. Thus, other reactions had to be occurring in the model systems containing vescalagin (model systems F and J), causing its greater disappearance.

Taking into account that all of the model systems were prepared in ethanol, compounds deriving from the reactions between ethanol and the different ellagitannins were also investigated in the HPLC–DAD–MS^*n*^ conditions employed for the analysis of ellagitannins. Two main types of ethanol-derived products have been reported to occur. First, Puech and co-workers^45^ were able to detect the derivative compounds formed from castalagin and vescalagin by the addition of ethoxy moieties to the structure of ellagitannin. Because this addition does not involve the chiral center at C1 of the ellagitannin structure, the resulting products still maintain the distinct conformation of this carbon, and this makes possible to detect derivatives coming from vescalagin and derivatives coming from castalagin at retention times greater than those of native ellagitannin. In contrast, the second type of derivatives that can be formed with ethanol involves C1 and can only be synthesized from vescalagin,^34,46^ giving rise to β-1-*O*-ethylvescalagin. Thus, in the present study, samples were screened to detect by XIC signals at the *m*/*z* ratios of β-1-*O*-ethylvescalagin (*m*/*z* 961, negative ion mode) and the *m*/*z* ratios of the ethoxy derivatives of vescalagin and castalagin (*m*/*z* 977, negative ion mode). No signals corresponding to β-1-*O*-ethylvescalagin were observed in any of the model systems. However, a signal at *m*/*z* 977 could be observed in all of the model systems containing vescalagin either alone (model systems C, F, and J) or with castalagin (model systems D, G, and K) but not in the model systems exclusively containing castalagin (Figure S4 of the Supporting Information). This means that, in the conditions of the present study, the formation of the ethoxy derivative of castalagin was not favored. Consequently, in the model systems containing vescalagin (C, F, and J), the greater reduction observed in the levels of ellagitannin in relation to those containing castalagin (B, E, and I; Figure 4) might be also attributed to the formation of the ethoxy derivative. Among the former model systems (C, F, and J), the lowest levels occurred where the synthesis of vitisin A was taking part (model system C; Figure S4 of the Supporting Information), meaning that, in relation to model systems F and J, a smaller proportion of vescalagin is devoted to the formation of this ethoxy derivative probably as a consequence of the participation of vescalagin in the synthesis of vitisin A. In the other two model systems (F and J), a similar formation could be observed.

In the model systems containing an equimolar mixture of castalagin and vescalagin (D, G, and K; Figure 4), faster and greater losses were observed for each ellagitannin in relation to those observed in the corresponding model systems containing only one of them. No greater synthesis of vitisin A (Figure 1b) or 10-methylpyranomalvidin 3-*O*-glucoside (Figure S2 of the Supporting Information) was observed in model system D in relation to model systems B or C that would explain the greater disappearance of ellagitannins. Similarly, no greater contents of degradation products of mv-3-glc could be observed in model system K in relation to model systems I and J. Consequently, the greater loss of ellagitannins can be mostly attributed to an increase of the ellagitannin degradation reactions promoted by the presence of the other ellagitannin in the same solution, as previously reported in simpler model systems.^28^ At day 122, as occurred in model systems with only one ellagitannin, the greatest decreases in the levels of both ellagitannins occurred in the model system containing pyruvic acid and mv-3-glc (model system D; Figure 4) (4.5% of the initial levels of castalagin and 0.08% of those of vescalagin), followed by that prepared only with mv-3-glc (model system K; 6.9% of castalagin and 1.5% of vescalagin) and that prepared with pyruvic acid (model system G; 17.5% of castalagin and 10% of vescalagin). Bearing in mind the values observed in the model systems containing only one ellagitannin, vescalagin seems to be more affected by the presence of castalagin than castalagin by the presence of vescalagin. These results are contrasting with those reported in the previous study carried out in simpler model systems,^28^ but differences in the behavior might be related to the fact that the model systems of the present study were prepared in wine-like solution and those of the previous study in ultrapure water. For instance, the presence of ethanol has made the formation of the ethoxy derivative of vescalagin in these model systems possible (D, G, and K; Figure S4 of the Supporting Information), causing a greater disappearance of vescalagin in relation to that observed in ultrapure water, where it could not be formed. In addition, because, in the conditions of the present study, the ethoxy derivative of castalagin was not formed, a relatively greater reduction in the levels of vescalagin was occurring. In turn, the presence of castalagin could have influenced the evolution of this ethoxy derivative, which showed a different behavior from that observed in the model systems exclusively containing vescalagin (C, F, and J; Figure S4 of the Supporting Information), with a fast formation during the first days but then staying quite stable. These results highlight the complexity of the interactions that can be occurring in wine, where a large variety of grape native anthocyanins can react with a large variety of compounds coming from grapes or from the fermentation processes and where ellagitannins extracted from the oak wooden containers can be modulating the extent of these reactions.

In summary, the results of the present study confirmed that mv-3-glc disappears much faster in the presence of pyruvic acid than in its absence. They also demonstrated that this faster disappearance in the presence of pyruvic acid was mainly due to the synthesis of vitisin A, whereas in the absence of pyruvic acid, degradation reactions of mv-3-glc prevailed. The presence of ellagitannins affected both types of reactions. In general, the synthesis of vitisin A was initially increased in the presence of castalagin or vescalagin, although its final content depended upon the type of ellagitannin. In contrast, in the case of the degradation products, the levels were more affected by the presence or absence of ellagitannins than by the ellagitannin type. In the absence of pyruvic acid and the presence of ellagitannins, the degradation product profile was modified and the total content of degradation products was reduced at the end of the experiment. Despite this lower formation of degradation products in the presence of ellagitannins, a greater disappearance of mv-3-glc could be observed in these model systems, pointing to a possible role of ellagitannins in favoring the synthesis of anthocyanin-derived pigments, even in the absence of pyruvic acid. The co-existence of more than one type of ellagitannins in the model systems also modified the levels of the individual ellagitannins, which could affect, in turn, the synthesis of vitisin A or the formation of degradation products. Although ellagitannins had been postulated in previous studies to play a relevant role in the synthesis of vitisin A, this work gives, for the first time, evidence of their role. Furthermore, it is the first time that the influence of these compounds in the degradation of mv-3-glc has been reported and studied. These results, obtained in model systems, can be very useful to understand the role of ellagitannins in anthocyanin transformation and degradation in red wines aged in oak barrels. In addition, they highlight the relevance that barrel aging can have in the stabilization of wine color and the importance of the type of oak wood and number of fillings of the barrels, because these factors directly condition the quantitative and qualitative ellagitannin composition of the wine.

## Supporting Information Available

Model systems prepared for the study of the formation of A-type vitisins and degradation products of malvidin 3-*O*-glucoside in the presence and absence of ellagitannins (Figure S1). Evolution of the levels of 10-methylpyranomalvidin 3-*O*-glucoside (expressed in mg/L of malvidin 3-*O*-glucoside) in the model systems containing malvidin 3-*O*-glucoside and pyruvic acid in the absence and presence of ellagitannins (model systems A, B, C, and D) (Figure S2). Evolution of the mean percentages of the main degradation compounds of malvidin 3-*O*-glucoside from days 1 to 122 in model systems H, I, J, and K and at day 122 in model systems A, B, C, and D (Figure S3). Evolution of the area of the peak corresponding to the ethoxy derivative of vescalagin (*m*/*z* 977) in the chromatograms recorded at 280 nm of the model systems containing ellagitannin(s), malvidin 3-*O*-glucoside, and pyruvic acid (model systems B, C, and D), ellagitannin(s) and pyruvic acid (model systems E, F, and G) or ellagitannin(s) and malvidin 3-*O*-glucoside (model systems I, J, and K) (Figure S4). Mean percentage of malvidin 3-*O*-glucoside remaining in the samples (Table S1). Mean content of vitisin A (mg/L) in the model systems containing pyruvic acid (model systems A, B, C, and D) (Table S2). Chromatographic and UV and mass spectral features of the main degradation peaks observed at day 122 in model system H in the chromatogram recorded at 280 nm (Table S3). Mean individual and total areas of the main degradation compounds determined in model systems H, I, J, and K (without pyruvic acid) from days 1 to 122 and in model systems A, B, C, and D (with pyruvic acid) at day 122 (Table S4), and mean percentages of the initial concentration of each ellagitannin in the model systems containing mv-3-glc, pyruvic acid, and one or two ellagitannins (model systems B, C, and D), in the model systems containing pyruvic acid and one or two ellagitannins (model systems E, F, and G), and in the model systems containing mv-3-glc and one or two ellagitannins (model systems I, J, and K) (Table S5) (PDF)
