# Supplementary material for: Role of Oak Ellagitannins in the Synthesis of Vitisin A and in the Degradation of Malvidin 3-O-Glucoside: An Approach in Wine-Like Model Systems
Source: J Agric Food Chem. 2022 Apr 19;70(41):13049–61. doi: 10.1021/acs.jafc.2c00615 (PMC9585584; doi:10.1021/acs.jafc.2c00615)
Supplement: Supplementary file 1 — jf2c00615_si_001.pdf [file jf2c00615_si_001.pdf]

SUPPLEMENTARY FILES

Role of oak ellagitannins in the synthesis of vitisin A and in the degradation of malvidin 3-*O*-glucoside. An approach in wine-like model systems.

Alcalde-Eon, Cristina*; Escribano-Bailón, María-Teresa; García-Estévez, Ignacio.

Grupo de Investigación en Polifenoles. Departamento de Química Analítica, Nutrición y Bromatología. Facultad de Farmacia. University of Salamanca. E-37003, Salamanca, Spain.

Tel: +34 923 29 45 37

e-mail addresses:

Alcalde-Eon, Cristina: crisalcaldeon@usal.es *corresponding author

Escribano-Bailón, María-Teresa: escriban@usal.es

García-Estévez, Ignacio: igarest@usal.es

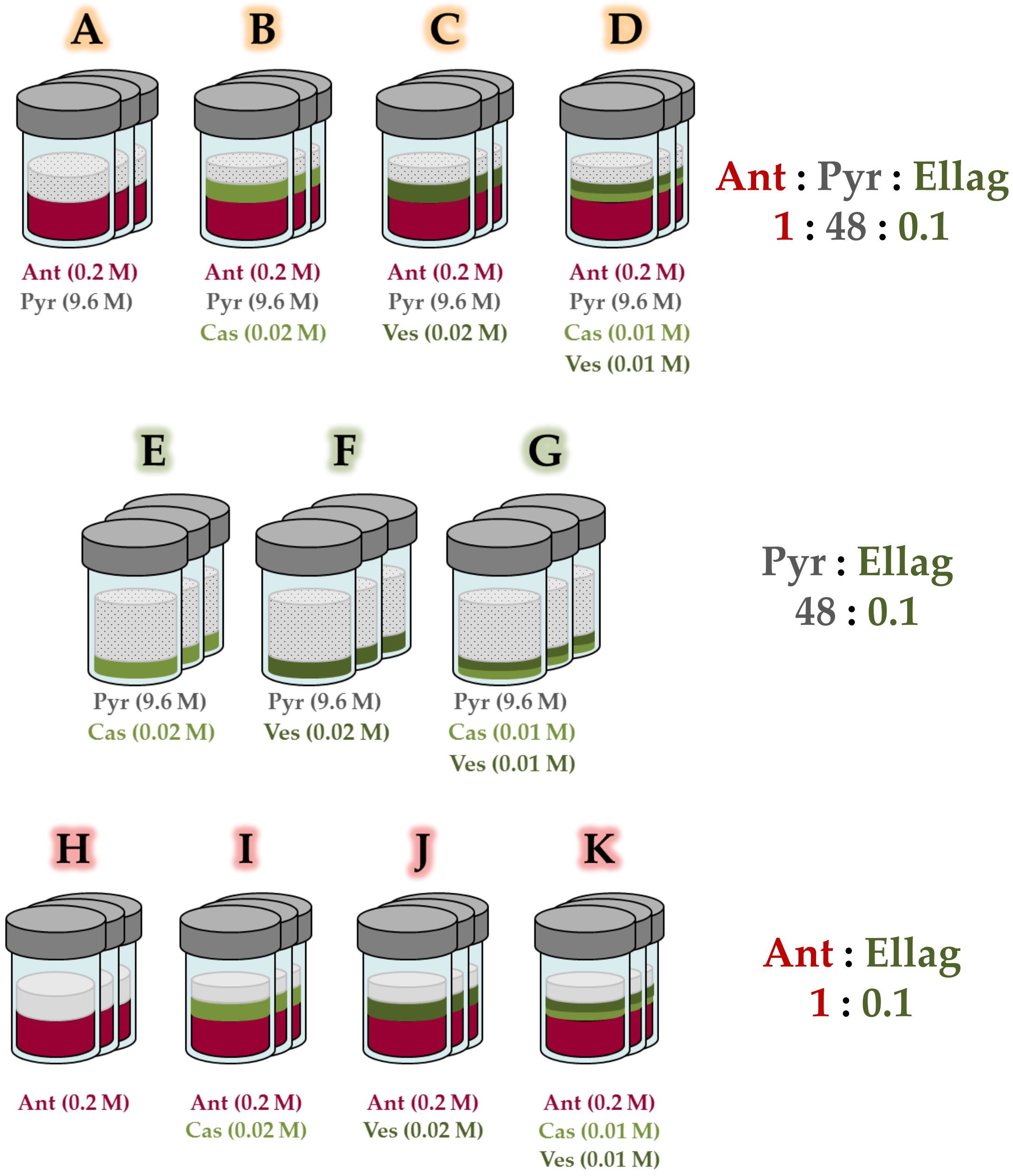

Figure S1. Model systems prepared for the study of the formation of A-type vitisins and degradation products of malvidin 3-*O*-glucoside in the presence and absence of ellagitannins. Ant: anthocyanins, Pyr: pyruvic acid; Ellag: ellagitannins; Cas: castalagin; Ves: vescalagin.

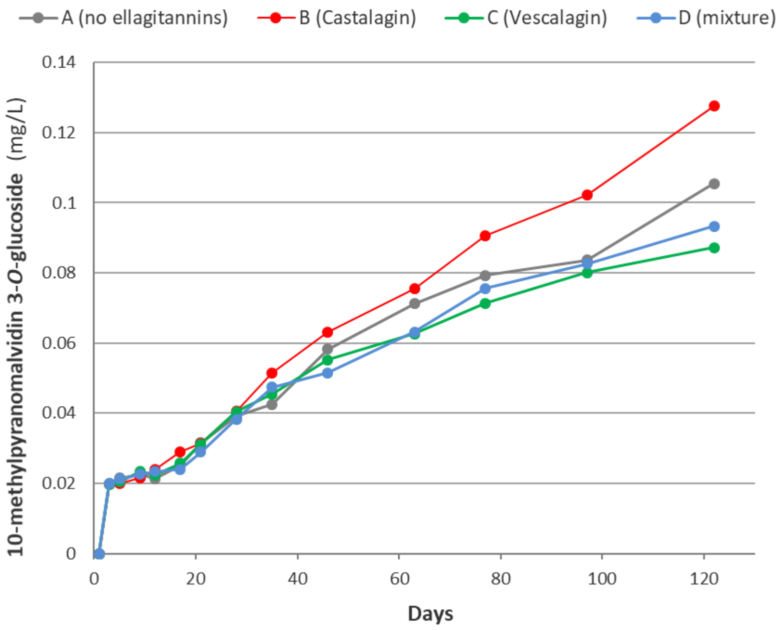

Figure S2. Evolution of the levels of 10-methylpyranomalvidin 3-*O*-glucoside (expressed in mg/L of malvidin 3-*O*-glucoside) in the model systems containing malvidin 3-*O*-glucoside and pyruvic acid in the absence and presence of ellagitannins (model systems A, B, C and D).

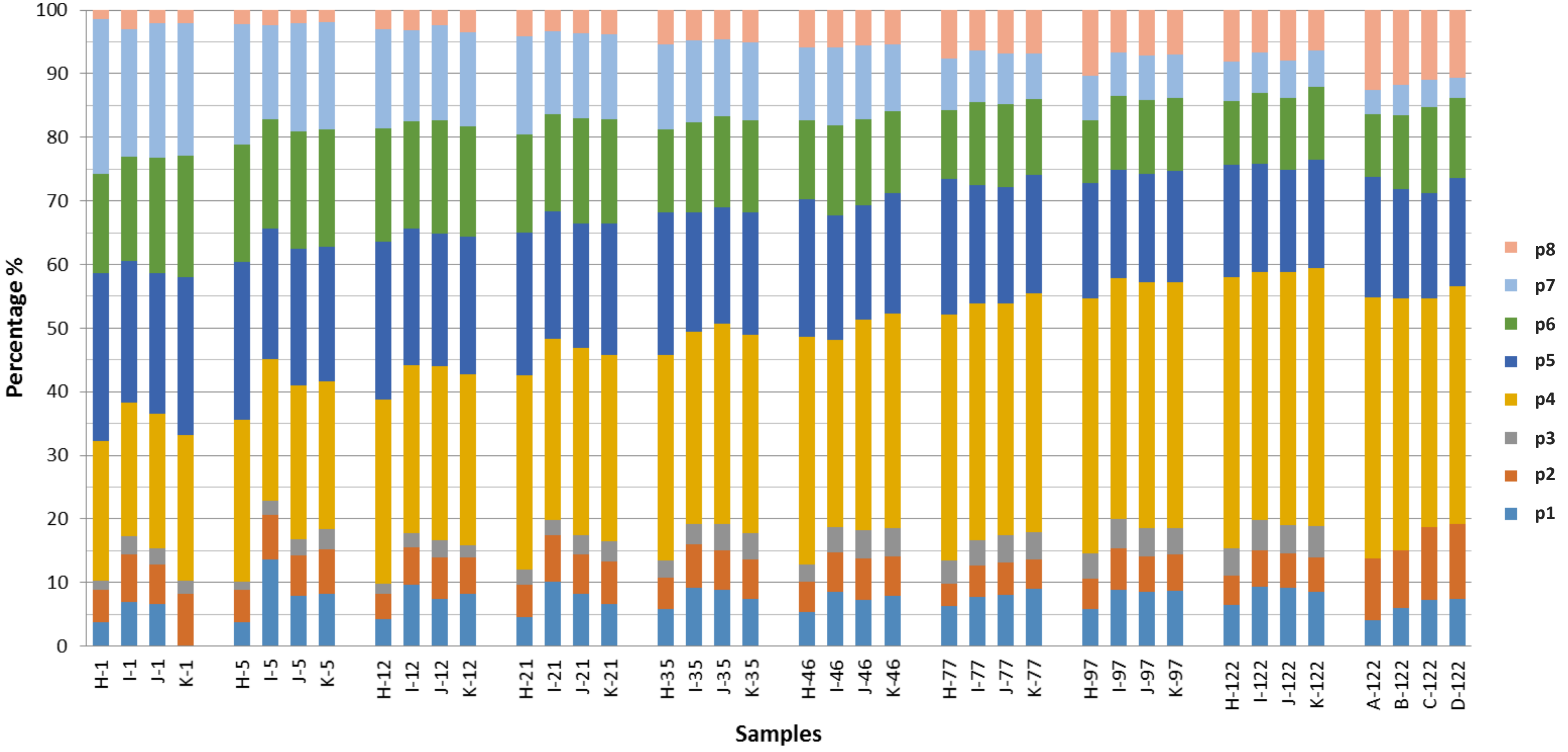

Figure S3. Evolution of the mean (n=3) percentages of the main degradation compounds (**p1**, **p2**, **p3**, **p4**, **p5**, **p6**, **p7** and **p8**) of malvidin 3-*O*-glucoside from day 1 to day 122 in model systems H, I, J and K. The mean percentages of these compounds in model systems A, B, C and D at day 122 are also shown for comparative purposes.

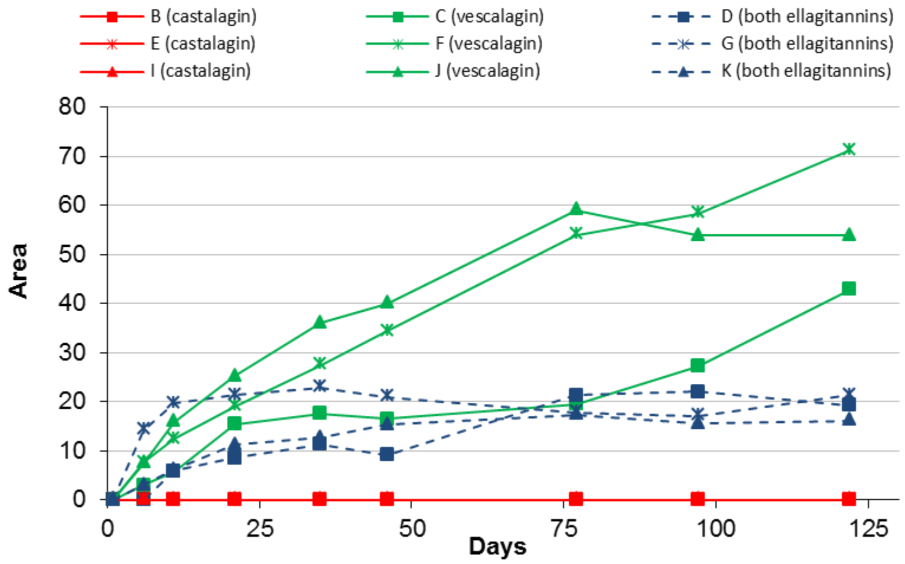

Figure S4. Evolution of the area of the peak corresponding to the ethoxy-derivative of vescalagin (*m/z* 977, negative ion mode) in the chromatograms recorded at 280 nm of the model systems containing ellagitannin(s), malvidin 3-*O*-glucoside and pyruvic acid (model systems B, C and D), ellagitannin(s) and pyruvic acid (model systems E, F and G) or ellagitannin(s) and malvidin 3-*O*-glucoside (model systems I, J and K).

Table S1. Mean (n=3) percentage of malvidin 3-*O*-glucoside remaining in the samples. in relation to the content at the beginning of the experiment (day 1).

	**Model systems**							
	with pyruvic acid				*without pyruvic acid*			
**Days**	**A**	**B**	**C**	**D**	**H**	**I**	**J**	**K**
1	100.00a	100.00a	100.00a	100.00a	100.00a	100.00a	100.00a	100.00a
3	92.61ab	94.74ab	95.03b	92.22a	98.62a	98.65a	100.00a	99.09a
5	87.56a	90.38b	90.71b	87.93a	98.55a	97.94a	99.04a	99.23a
9	75.89a	78.43b	79.83c	75.31a	96.22a	96.78a	97.39a	96.41a
12	69.09a	72.87b	73.92b	69.72a	96.95a	95.83a	96.43a	96.54a
17	60.02a	63.14b	63.96b	60.94a	95.73a	93.37a	94.32a	93.48a
21	54.79a	55.69b	57.60c	54.71a	92.26a	92.54a	91.21a	92.51a
28	48.35a	47.27a	51.36a	48.74a	92.15a	90.46a	88.38a	90.45a
35	42.18a	44.74b	45.46b	42.73a	89.31a	86.64a	86.22a	87.15a
46	34.95a	36.88b	38.22b	34.62a	80.51a	81.44a	82.37a	80.81a
63	28.66a	30.49b	31.75c	28.91a	79.43a	73.07a	71.89a	73.12a
77	25.03a	26.54bc	27.63c	25.90ab	76.00b	69.19a	67.47a	69.44a
97	19.92a	21.94ab	23.28b	21.89ab	71.27b	64.02a	62.30a	62.95a
122	15.31a	15.68a	17.30b	16.83b	61.01c	53.33b	50.70a	51.78ab

Except for day 1, results from model systems containing pyruvic acid were statistically different (p<0.05) from those from model systems built without pyruvic acid. Different letters in the same row indicate statistically significant differences (p<0.05) among models systems of a same type (with or without pyruvic acid).

Table S2. Mean content (n=3) of vitisin A (mg/L) in the model systems containing pyruvic acid (model systems A, B, C and D).

	**Model systems**			
**Days**	**A**	**B**	**C**	**D**
1	0.00a	0.00a	0.00a	0.00a
3	0.62a	0.73b	0.74b	0.75b
5	1.03a	1.11b	1.02a	1.02a
9	1.47a	1.58b	1.52ab	1.54ab
12	1.79a	1.92b	1.82a	1.81a
17	2.19a	2.40b	2.21a	2.27ab
21	2.50a	2.66a	2.58a	2.57a
28	2.92a	2.99a	2.96a	2.88a
35	3.20a	3.58b	3.29ab	3.22a
46	3.53a	3.91b	3.58a	3.34a
63	4.06a	4.70b	3.98a	3.82a
77	4.61a	5.27b	4.36a	4.22a
97	4.93a	5.99b	4.72a	4.47a
122	5.70b	6.54c	4.85a	4.68a

Different letters in the same row indicate significant differences ($p<0.05$).

Table S3. Chromatographic and UV and mass spectral features of the main degradation peaks observed at day 122 (see Figure 2) in model system H in the chromatogram recorded at 280 nm. The identities proposed for them are also indicated.

**Peak**	t_R (min)	λ_{max} (nm)	$[M^+]$ (*m/z*)	MS^2 fragment Ions (*m/z*)	Identity
**p1**	7.1	294, 336 (sh)	171		2,4,6-trihydroxybenzoic acid
**p2**	9.9	302			
**p3**	16.0	290	169		
**p4**	19.0	274	199		Syringic acid
**p5**	22.7	292, 336 (sh)	155		2,4,6-trihydroxybenzaldehyde
**p6**	28.5	294, 330 (sh)	335	181	(see ref. 43 for structure)
**p7**	32.8	344	511	349	Chalcone 3-*O*-glucoside of mv
**p8**	40.2	282	183		Syringaldehyde

(mv: malvidin; sh: shoulder).

Table S4. Mean (n=3) individual and total areas of the main degradation compounds determined in model systems H, I, J and K (without pyruvic acid) from day 1 to day 122 and in model systems A, B, C and D (with pyruvic acid) at day 122.

	**Model systems H, I, J and K**									**A, B, C and D**	
**Days**	**1**	**5**	**12**	**21**	**35**	**46**	**77**	**97**	**122**		**Day 122**
**Peak p1**											
H	42.9b	49.8a	61.1a	77.7a	112.2a	103.8a	167.5a	180.6a	231.4aαβ	A	143.6α
I	83.1d	190.7c	141.7d	172.2c	174.0c	169.1b	193.4b	252.1b	299.8aβ	B	226.2αβ
J	74.6c	104.5b	102.6b	133.3b	167.4bc	152.3b	202.1b	240.3b	287.2aβ	C	213.4αβ
K	0.0a	114.5b	122.2c	114.3b	145.0b	162.4b	219.1b	241.4b	263.7aβ	D	223.5αβ
**Peak p2**											
H	59.8a	65.7a	57.0a	84.0a	94.8a	92.3a	93.6a	150.0a	171.3aα	A	323.7β
I	87.7ab	97.1b	87.1b	122.9c	131.4b	121.4ab	122.7a	185.8c	186.8aα	B	336.0β
J	70.9ab	82.9b	91.9b	99.2b	119.1ab	133.8b	125.5a	157.7b	170.2aα	C	340.4β
K	91.1b	97.7b	86.5b	112.1c	120.8ab	126.4b	108.2a	160.4b	161.1aα	D	360.5β
**Peak p3**											
H	17.0a	17.2a	24.2a	38.5a	53.8a	52.5a	96.2a	123.8a	152.0ab	A	n.d.
I	34.7b	30.2b	33.1b	41.2a	60.4a	79.3b	97.3a	132.5a	154.4b	B	n.d.
J	29.5ab	32.9b	36.8b	49.7b	77.1b	91.0b	106.4a	123.7a	141.9a	C	n.d.
K	22.8ab	45.6c	27.3a	55.6b	78.7b	91.1b	105.5a	114.3a	155.2b	D	n.d.
**Peak p4**											
H	253.6c	331.0a	418.9b	510.9a	624.5a	690.9a	1028.2b	1248.1b	1541.6bδ	A	1387.6γ
I	248.1b	310.6a	391.9ab	478.5a	572.1a	584.9a	923.2a	1085.3a	1253.3aβ	B	1463.3γδ
J	240.5a	318.2a	382.8a	478.3a	594.6a	685.1a	907.7a	1085.2a	1247.8aβ	C	1064.7α
K	254.4c	323.8a	400.5ab	496.9a	605.5a	687.7a	905.8a	1068.1a	1237.1aβ	D	1135.2αβ
**Peak p5**											
H	305.2d	323.0b	360.6b	376.4b	430.7b	415.7b	562.4b	568.9b	640.1cβ	A	642.0β
I	263.0b	286.9a	317.8a	340.6ab	358.6a	386.8a	460.7a	484.4a	548.1bα	B	637.6β
J	251.1a	283.3a	290.6a	314.4a	347.6a	374.7a	457.8a	475.2a	502.8aα	C	489.5α
K	274.4c	294.9ab	321.5a	354.9ab	374.7a	383.1a	446.3a	484.9a	523.3aα	D	517.7α
**Peak p6**											
H	179.5a	239.1a	258.5b	259.8a	253.8a	241.9a	290.6a	306.6a	364.7aαβ	A	334.4α
I	192.2a	238.2a	249.2a	257.3a	269.6b	278.3b	322.3a	332.7b	358.2aαβ	B	432.1γ
J	206.5a	242.8a	246.3a	269.4a	270.5b	277.9b	322.4a	326.9b	354.7aαβ	C	397.6βγ
K	211.0a	257.6a	258.6b	278.6a	281.5b	263.0ab	287.6a	319.0ab	351.9aα	D	377.9αβ
**Peak p7**											
H	281.9b	247.9b	225.7a	257.5a	257.2b	220.7a	215.1b	219.6b	221.8cδ	A	130.3β
I	237.0a	206.0a	212.5a	218.0a	243.2ab	244.3b	203.4ab	196.6ab	205.8bcγδ	B	174.2γ
J	240.1a	223.3ab	208.9a	215.9a	226.4a	242.9b	198.6ab	196.4ab	187.0abγ	C	127.4β
K	230.5a	234.7b	221.3a	228.8a	238.2ab	214.9a	174.8a	187.7a	173.6aγ	D	96.2α
**Peak p8**											
H	17.2a	28.1a	43.4ab	70.0b	102.9a	113.1a	202.5a	318.9b	295.1cβγ	A	430.0δ
I	36.7a	33.6a	46.4b	57.4a	91.4a	115.5a	155.8a	188.6a	212.9aα	B	436.0δ
J	23.3a	27.0a	33.7a	59.6a	88.5a	114.1a	171.9a	199.2a	247.8bαβ	C	324.4γ
K	23.7a	26.9a	51.0b	63.7ab	97.6a	110.1a	163.5a	194.8a	194.3aα	D	326.1γ
**TOTAL**											
H	1157.0a	1301.6a	1449.2a	1674.7a	1929.8a	1930.7a	2656.0a	3116.2b	3617.8cγ	A	3391.5βγ
I	1182.2a	1393.1b	1479.5a	1687.8a	1900.6a	1979.3a	2478.5a	2858.0a	3219.2bαβ	B	3705.2γ
J	1136.3a	1314.8a	1393.5a	1619.6a	1891.0a	2071.6a	2492.3a	2804.4a	3139.2abαβ	C	2957.3α
K	1107.7a	1395.6b	1488.8a	1704.7a	1941.9a	2038.5a	2410.7a	2770.3a	3060.0aαβ	D	3037.0αβ

Different roman letters in the same column for a given compound indicate significant differences (p<0.05). Greek letters are used for the comparison between model systems containing pyruvic acid and those where it was absent at day 122 (p<0.05).

Table S5. Mean percentages (n=3) of the initial concentration of each ellagitannin in the model systems containing malvidin 3-*O*-glucoside (mv-3-glc), pyruvic acid and castalagin (Cas), vescalagin (Ves) or both (model systems B, C and D), in the model systems containing pyruvic acid and one or two ellagitannins (model systems E, F and G) and in the model systems containing mv-3-glc and one or two ellagitannins (model systems I, J and K). From these percentages, the graphs shown in Figure 4 (4a, 4b and 4c, respectively) have been built.

	Model systems			
**Day**	**B_Cas**	**D_Cas**	**C_Ves**	**D_Ves**
1	100.00a	100.00a	100.00a	100.00a
6	96.82c	90.53b	89.29b	83.82a
11	85.61c	80.54bc	77.39ab	72.06a
21	70.85b	64.43b	64.78b	48.29a
35	41.54c	36.46b	41.15c	20.10a
46	25.79c	19.50b	23.80c	7.19a
77	16.90c	11.70b	5.23a	2.24a
97	11.67c	6.46b	2.26a	0.92a
122	5.79b	4.51b	0.38a	0.07a
**Day**	**E_Cas**	**G_Cas**	**F_Ves**	**G_Ves**
1	100.00a	100.00a	100.00a	100.00a
6	96.50b	90.41a	92.85ab	91.35a
11	95.10c	85.97ab	86.80b	82.39a
21	86.72c	76.75b	78.94b	67.27a
35	73.31c	60.73b	64.89b	49.60a
46	64.86c	51.57b	56.13b	34.85a
77	47.37d	33.21b	36.93c	22.53a
97	38.71d	25.33b	30.17c	14.80a
122	27.80d	17.55b	21.34c	10.14a
**Day**	**I_Cas**	**K_Cas**	**J_Ves**	**K_Ves**
1	100.00a	100.00a	100.00a	100.00a
6	95.20c	88.91b	87.75ab	85.73a
11	89.97a	81.90a	83.11a	79.76a
21	80.63c	67.06a	73.53b	64.63a
35	64.70d	45.67b	50.55c	40.57a
46	53.29c	34.77a	40.55b	31.64a
77	34.41d	20.09b	26.70c	13.47a
97	24.55c	13.13b	14.81b	4.84a
122	17.36c	6.88b	7.41b	1.49a

Different letters in the same row indicate significant statistical differences ($p<0.05$)
